# Case Report: First report of a Wilms tumor in an individual with Dias–Logan syndrome (*BCL11A*-related intellectual disability)

**DOI:** 10.3389/fonc.2025.1585492

**Published:** 2025-07-23

**Authors:** Alexandre G. Troullioud Lucas, Elise Fiala, Ahmed Razeq, Talia Sauerhaft, Anita P. Price, Juan Miguel Mosquera, Jeremy Miyauchi, Ming Gao, Michael F. Walsh, Michael V. Ortiz

**Affiliations:** ^1^ Department of Pediatrics, Memorial Sloan Kettering Cancer Center, New York, NY, United States; ^2^ Baylor College of Medicine, Children’s Hospital of San Antonio, San Antonio, TX, United States; ^3^ Department of Radiology, Memorial Sloan Kettering Cancer Center, New York, NY, United States; ^4^ Department of Pathology and Laboratory Medicine, Weill Cornell Medicine, New York, NY, United States; ^5^ Department of Pathology and Laboratory Medicine, Northwell Health Northern Westchester Hospital, Mount Kisco, NY, United States; ^6^ Clinical Sciences, Global Development, Regeneron Pharmaceuticals, Inc., Tarrytown, NY, United States

**Keywords:** case report, Wilms tumor, genetics, *BCL11A*, Dias–Logan syndrome

## Abstract

Dias–Logan syndrome (DLS) is a rare condition caused by heterozygous germline *BCL11A* pathogenic variants associated with global developmental delay, distinctive facial features, and asymptomatic persistence of fetal hemoglobin. There has been no evidence of an association between DLS and increased risk of cancer. We report the first instance of a child with DLS diagnosed with cancer, a Wilms tumor (WT), who is notably much older than the typical onset. Although this case alone is insufficient to warrant routine WT screening in DLS, given the extreme rarity, we cannot rule out an association with DLS and WT predisposition.

## Introduction

Dias–Logan syndrome (DLS), also known as *BCL11A*-related intellectual disability (*BCL11A*-ID), is caused by germline heterozygous pathogenic variants in *BCL11A*, which is located on chromosome 2p16.1 (1). DLS/*BCL11A*-ID is inherited in an autosomal dominant manner, most often caused by a *de novo* mutation. This is a very rare event, and only 75 individuals have been reported with germline pathogenic/likely pathogenic variants in *BCL11A*, with a median age at molecular diagnosis of 7 years (range 1–19 years). Patients, usually children, with DLS/*BCL11A*-ID may present with a variety of clinical manifestations including global developmental delay/intellectual disability of variable degree, neonatal hypotonia, microcephaly, non-specific dysmorphic facial features, behavior problems, autism spectrum disorder, and asymptomatic persistence of fetal hemoglobin. Some patients have also been reported to have growth delay, seizures, and gastrointestinal and musculoskeletal problems (2). Notably, there are no reports of individuals with DLS/*BCL11A*-ID diagnosed with either solid or hematologic malignancies, although there was one patient with a benign (and relatively common) osteochondroma (3).

We describe the first case of a child with DLS/*BCL11A*-ID diagnosed with Wilms tumor (WT), a rare pediatric embryonal tumor of the kidney with approximately 500 cases per year diagnosed in the United States (4–6). The mean age at diagnosis is 44 months for unilateral cases and 31 months for bilateral cases of Wilms tumor, and 75% of patients are diagnosed before 5 years of age (5). Although most cases occur apparently sporadically, 10%–20% of children with WT are noted to have an established cancer predisposition (7, 8).

## Case description

This case report describes a girl born to a non-consanguineous couple of Northern and Eastern European descent who was noted as an infant to have hypotonia and global developmental delays, including speech apraxia. Genetic testing was pursued including a chromosomal microarray, expanded neurodevelopmental gene panel, and trio exome sequencing. The microarray showed a copy number gain on 9q31.1 that was found to be maternally inherited and therefore reclassified as benign. Exome sequencing identified a *de novo* heterozygous likely pathogenic germline variant denoted *BCL11A* c.55 + 1G>A. The genomic and clinical history was consistent with DLS*/BCL11A*-ID.

**Figure 1:**
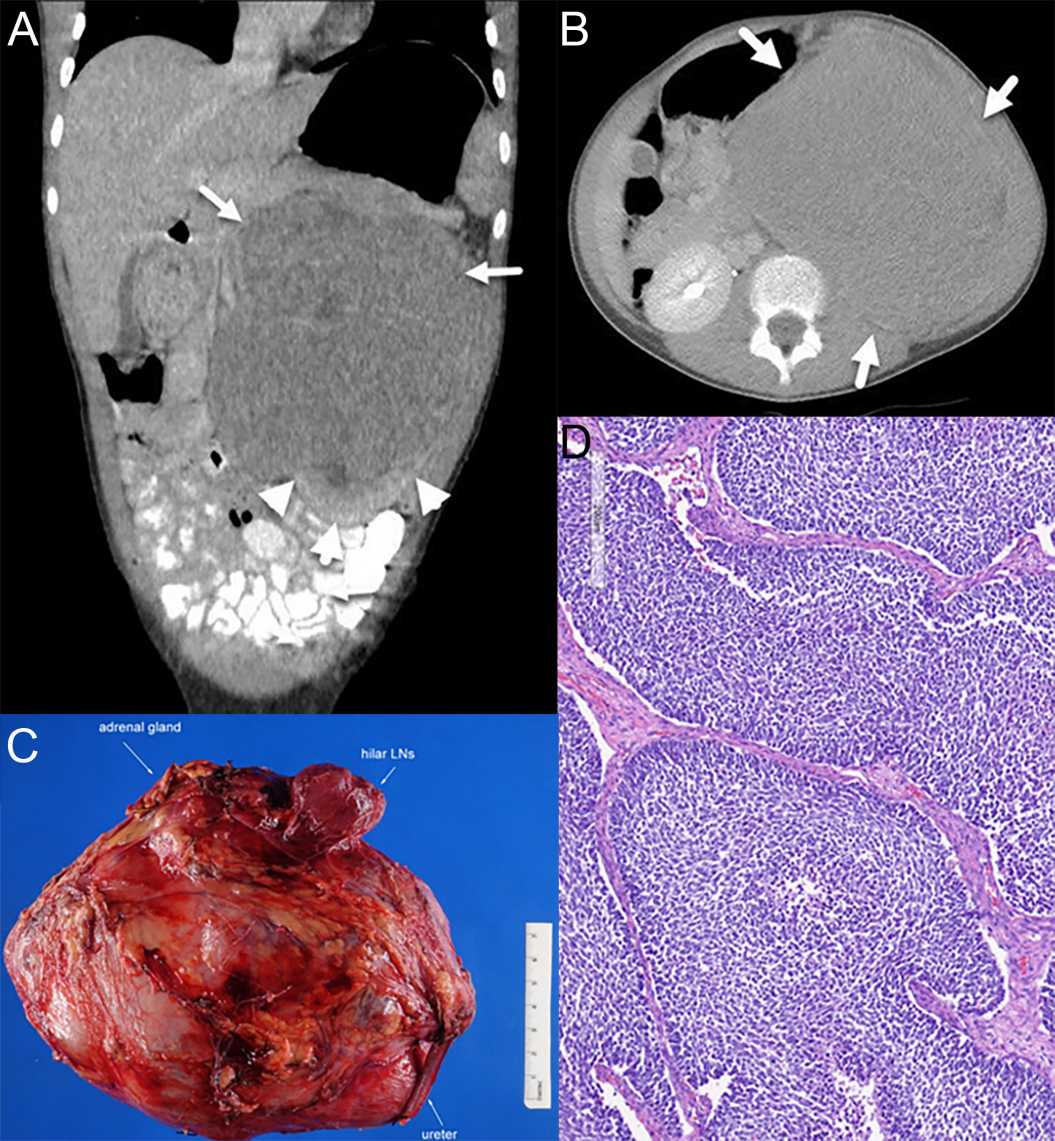
Radiology and pathology of the patient’s tumor. **(A)** Coronal view on CT scan at diagnosis. Arrows indicate tumor, and arrowheads show normal kidney and claw sign, demonstrating the renal origin. **(B)** Axial view on CT scan at diagnosis. **(C)** Gross appearance exhibiting a large renal mass. **(D)** Representative histology of the Wilms tumor with predominantly blastemal elements.

At 11 years old, she developed increasing episodes of headache and associated eye pain along with nausea and vomiting that awoke her from sleep. The left side of her abdomen was tender and distended. She was directed to a local emergency room, where an abdominal ultrasound identified a large left-sided renal lesion with mass effect on the infrarenal aorta. A CT scan of her chest, abdomen, and pelvis identified a 14-cm heterogeneous left renal mass without evidence of distant metastatic spread (**Figures 1A, B**). She underwent upfront left complete nephrectomy, and surgical pathology was consistent with a favorable histology Wilms tumor, predominantly blastemal in appearance, local stage III due to lympho-vascular invasion of the tumor with multiple regional lymph nodes demonstrating evidence of tumor (**Figures 1C, D**). The possibility of an additional condition resulting in a cancer predisposition was evaluated using germline Memorial Sloan Kettering-Integrated Mutation Profiling of Actionable Cancer Targets (MSK-IMPACT), which analyzed 90 cancer predisposition genes, as well as dedicated testing for Beckwith–Wiedemann syndrome (BWS), including methylation and copy number analysis of 11p15.5 and *CDKN1C* sequencing. No established cancer predisposition syndrome was identified through these tests. There were no potential environmental exposures that we could identify as contributors to cancer development, such as certain maternal exposures to carcinogens during pregnancy. The patient does not have a family history of childhood cancer and has multiple healthy siblings.

Cytogenetic testing of the tumor was positive for loss of heterozygosity (LOH) of chromosome 1p, but negative for LOH of chromosome 16q and negative for copy number gain of chromosome 1q. On Children’s Oncology Group (COG) Study AREN0532, the presence of LOH 1p and positive lymph node status was noted to be an adverse prognostic finding with a 4-year event-free survival of 73.8% compared to 88% for the stage III study cohort as a whole and 96.7% for stage III patients without LOH 1p nor 16q and no lymph node involvement (9). Additionally, her older age of onset along with somatic *DROSHA* miRNA microprocessing defect was further concerning (10, 11). Using shared decision making with the family based on these results, the decision was made to augment chemotherapeutic treatment from Regimen DD4A to Regimen M, thereby incorporating cyclophosphamide and etoposide onto her existing vincristine, dactinomycin, and doxorubicin backbone as per the COG Study AREN0533. She finished treatment, including standard dose flank radiation therapy, without any unexpected adverse events, and is now more than 3 years from completion of therapy with no evidence of disease recurrence.

## Discussion

This is the first report of a child with DLS/*BCL11A*-ID diagnosed with a malignancy, in this case, Wilms tumor, a rare pediatric kidney tumor. Currently, there is no evidence that individuals with DLS/*BCL11A*-ID are at an increased risk of developing cancer. However, as there are so few cases reported, and at such young average ages, it is possible that a low-penetrant cancer predisposition may be as-yet unknown (3). Notably, while there are no known reports of WTs in individuals with germline deletions or duplications involving *BCL11A* (2p15p16.1 deletion or duplication syndrome), renal anomalies such as multicystic kidneys and hydronephrosis have been reported in this related condition, suggesting that perhaps inactivation of BCL11A may be involved in physiologic renal development, although these syndromes include many additional adjacent genes, in addition to *BCL11A*, which may instead be contributory (12).

The established function of BCL11A is that it forms a key component of the mammalian SWI/SNF complex, a chromatin remodeling apparatus that has been implicated in 20% of all human tumors and notably aberrant in several other pediatric kidney tumors such as malignant rhabdoid tumor of the kidney and renal medullary carcinoma, both of which are characterized by biallelic inactivation of *SMARCB1* (13); indeed, somatic alterations of *BCL11A* are noted in many different, generally adult-onset, cancers (14). While we are unaware of genetic somatic inactivation of *BCL11A* as a driver of Wilms tumors, *BCL11A* is indeed one of the most highly downregulated transcripts in *SIX1*-mutant WT, which notably form a related subset of WT characterized by a pre-induction metanephric mesenchyme gene expression profile and often associated with *DROSHA* mutations, as seen in this case (10, 15). Along with other key epigenomic drivers of nephrogenesis, including other SWI/SNF complex constituents or downstream PRC2 complex components, BCL11A appears to play a role in the differentiation program of nephron progenitor cells, further supporting its potential as a tumor suppressor candidate (16).

In children diagnosed with WT predisposition conditions, surveillance typically involves renal ultrasonography every 3–4 months until age 7 (17). The patient described in this case was diagnosed at 11 years of age, so she would not have been identified earlier with conventional screening strategies for established WT cancer predisposition syndromes. In the most common WT predisposition condition, BWS, WT screening has been shown to result in an earlier stage at diagnosis of tumors. Given the importance of *BCL11A* in cancer, and this case report, it will be important to determine over time if other individuals with DLS/*BCL11A*-ID are diagnosed with WT or other cancer types, if screening is necessary, and what age range of screening would be most appropriate (18). As such, we do feel that this very rare constellation of possibly related conditions is worth describing. Indeed, as selective knockdown of *BCL11A* has become a novel therapeutic strategy to treat sickle cell disease, a further understanding of this gene will become more important beyond rare cases of children with DLS/*BCL11A*-ID (19).

In conclusion, we describe an 11-year-old female patient with DLS/*BCL11A*-ID who developed WT, and we raise the possibility that DLS/*BCL11A*-ID could be associated with WT predisposition, although further observations are needed to better characterize this association, if any. At this point, there are inadequate data to warrant screening for WT in patients with DLS/*BCL11A*-ID, but future research should focus on what tumor spectrum, if any, is associated with this condition in order to design an appropriate screening strategy.

## Data Availability

The raw data supporting the conclusions of this article will be made available by the authors, without undue reservation.
